# S100 proteins as potential predictive biomarkers of abatacept response in polyarticular juvenile idiopathic arthritis

**DOI:** 10.1186/s13075-024-03347-0

**Published:** 2024-06-25

**Authors:** Hermine I Brunner, Grant S Schulert, Alyssa Sproles, Sherry Thornton, Gabriel Vega Cornejo, Jordi Antón, Ruben Cuttica, Michael Henrickson, Ivan Foeldvari, Daniel J Kingsbury, Margarita Askelson, Jinqi Liu, Sumanta Mukherjee, Robert L Wong, Daniel J Lovell, Alberto Martini, Nicolino Ruperto, Alexei A Grom, on behalf of the Pediatric Rheumatology Collaborative Study Group (PRCSG) and the Paediatric Rheumatology International Trials Organisation (PRINTO)

**Affiliations:** 1 Division of Rheumatology, Department of Pediatrics, Cincinnati Children’s Hospital Medical Center, University of Cincinnati College of Medicine, Cincinnati, OH USA; 2 Hospital México Americano, Guadalajara, CREA Mexico; 3 Pediatric Rheumatology Department, Hospital Sant Joan de Déu, Universitat de Barcelona, Barcelona, Spain; 4 Ruben Cuttica MD, Pediatric Rheumatology, Hospital General de Niños Pedro de Elizalde, Buenos Aires, Argentina; 5 Hamburg Centre for Pediatric and Adolescent Rheumatology, Schön Klinik Hamburg Eilbek, Hamburg, Germany; 6 Division of Rheumatology, Randall Children’s Hospital at Legacy Emanuel, Portland, OR USA; 7 Global Biometric Sciences, Bristol Myers Squibb, Princeton, NJ USA; 8 Translational Medicine, Bristol Myers Squibb, Princeton, NJ USA; 9 Bristol Myers Squibb, Immunology and Fibrosis, Princeton, NJ USA; 10 Dipartimento di Neuroscienze, Riabilitazione, Oftalmologia, Genetica e Scienze Materno-Infantili (DiNOGMI), Università degli Studi di Genova, Genoa, Italy; 11 IRCCS Istituto Giannina Gaslini, Gaslini Trial Centre/Servizio di Sperimentazioni Cliniche Pediatriche, PRINTO, Genoa, Italy

**Keywords:** Abatacept, Biomarkers, C-Reactive protein (CRP), Juvenile idiopathic arthritis, S100

## Abstract

**Background:**

Juvenile idiopathic arthritis (JIA) comprises a heterogeneous group of conditions that can cause marked disability and diminished quality of life. Data on predictors of clinical response are insufficient to guide selection of the appropriate biologic agent for individual patients. This study aimed to investigate the propensity of S100A8/9 and S100A12 as predictive biomarkers of abatacept response in polyarticular-course juvenile idiopathic arthritis (pJIA).

**Methods:**

Data from a phase 3 trial (NCT01844518) of subcutaneous abatacept in patients with active pJIA (*n* = 219) were used in this exploratory analysis. Association between biomarker levels at baseline and improvements in JIA-American College of Rheumatology (ACR) criteria responses or baseline disease activity (measured by Juvenile Arthritis Disease Activity Score in 27 joints using C-reactive protein [JADAS27-CRP]) were assessed. Biomarker level changes from baseline to month 4 were assessed for disease outcome prediction up to 21 months.

**Results:**

At baseline, 158 patients had available biomarker samples. Lower baseline S100A8/9 levels (≤ 3295 ng/mL) were associated with greater odds of achieving JIA-ACR90 (odds ratio [OR]: 2.54 [95% confidence interval (CI): 1.25–5.18]), JIA-ACR100 (OR: 3.72 [95% CI: 1.48–9.37]), JIA-ACR inactive disease (ID; OR: 4.25 [95% CI: 2.03–8.92]), JADAS27-CRP ID (OR: 2.34 [95% CI: 1.02–5.39]) at month 4, and JIA-ACR ID (OR: 3.01 [95% CI: 1.57–5.78]) at month 16. Lower baseline S100A12 levels (≤ 176 ng/mL) were associated with greater odds of achieving JIA-ACR90 (OR: 2.52 [95% CI: 1.23–5.13]), JIA-ACR100 (OR: 3.68 [95% CI: 1.46–9.28]), JIA-ACR ID (OR: 3.66 [95% CI: 1.76–7.61]), JIA-ACR90 (OR: 2.03 [95% CI: 1.07–3.87]), JIA-ACR100 (OR: 2.14 [95% CI: 1.10–4.17]), and JIA-ACR ID (OR: 4.22 [95% CI: 2.15–8.29]) at month 16. From baseline to month 4, decreases in S100A8/9 and S100A12 generally exceeded 50% among JIA-ACR90/100/ID responders.

**Conclusion:**

Lower baseline levels of S100A8/9 and S100A12 proteins predicted better response to abatacept treatment than higher levels and may serve as early predictive biomarkers in pJIA. Decreases in these biomarker levels may also predict longer-term response to abatacept in pJIA.

**Supplementary Information:**

The online version contains supplementary material available at 10.1186/s13075-024-03347-0.

## Introduction

Juvenile idiopathic arthritis (JIA) is a heterogeneous group of conditions defined by chronic non-infectious arthritis persisting for 6 weeks or more, with onset in patients aged < 16 years [1]. If the inflammatory process is not swiftly controlled, JIA has the propensity to cause marked disability from joint damage, chronic pain, limitation of physical function, and diminished quality of life [1, 2].

In agreement with the treatment recommendations from an international task force of pediatric rheumatologists [3], the current American College of Rheumatology (ACR) guidelines recommend that most patients with a polyarticular course of JIA (pJIA) be initially treated with synthetic disease-modifying antirheumatic drugs (DMARDs), such as methotrexate (MTX) [4]. If disease activity persists or if there is intolerance to MTX, a biologic DMARD should be introduced. However, there are insufficient data available on predictors of clinical response to guide selection of the appropriate biologic agent for individual patients. Unless there is lack of tolerability, it is recommended that a biologic DMARD be used in conjunction with MTX for potential synergy [4]. Abatacept, a selective CD80/86 co-stimulation modulator that inhibits T-cell activation and impairs antigen presentation to T cells, is effective and well tolerated when used either intravenously or subcutaneously (SC) for the treatment of pJIA [5–7].

Despite evidence-based guidance, recommended treatment strategies for pJIA have a risk of failure; one study reported that 56–66% of patients with JIA receiving ≥ 5 biologic DMARDs continue to have chronically uncontrolled JIA [8]. Reliable, validated serum biomarkers of treatment response in JIA are needed to guide clinical decisions to allow patients to be treated with the most effective therapy as early as possible [9]. In particular, a high proportion of patients with JIA show incomplete response to MTX [10]. As such, strategies of combining biomarkers with options to treat JIA have been proposed [9]. Prior research shows that S100 proteins [11], specifically S100A8/9 (formerly MRP8/14 or calprotectin) and S100A12 (formerly calgranulin C or EN-RAGE), may be predictive biomarkers of treatment response in rheumatic diseases, including pJIA [12–14]. Serum S100A8/9 and S100A12 are alarmin proteins predominantly released at inflammatory sites by activated innate immune effectors including monocytes/macrophages and neutrophils [14, 15]; these proteins reflect the degree of local inflammation (e.g. synovitis) and are considered to be more specific biomarkers than other systemic inflammatory biomarkers such as C-reactive protein (CRP) and erythrocyte sedimentation rate (ESR) in rheumatoid arthritis (RA) [16, 17]. Prior studies suggested that S100A8/9 levels are associated with disease activity in patients with RA [12, 16, 17] and JIA [13, 14]. S100A12 has also been linked to autoimmune diseases, with strong expression found in inflamed tissues of adult patients with chronic arthritis [18]. Additionally, several studies have suggested that high levels of S100A8/9 and S100A12 are associated with response to both MTX and tumor necrosis factor (TNF) inhibitors [19–21]. However, whether baseline levels of S100 proteins predict differential treatment response to abatacept in pJIA remains unknown.

A phase 3 study in patients with active pJIA showed that weight-stratified SC abatacept treatment yielded target therapeutic exposures at month 4 (primary endpoint) and month 24, was well tolerated, and improved symptoms over a period of 24 months [6]. Here, we present an exploratory analysis using data from the phase 3 study to evaluate the potential of pre-treatment serum levels of S100A8/9 and S100A12 to serve as predictive biomarkers of clinical response to abatacept. We hypothesized that in a heterogeneous group of patients with JIA, those with low S100 levels may respond best to abatacept treatment.

## Patients and methods

### Patients and study design

This exploratory analysis includes data from a phase 3, single-arm, open-label, international, multicenter, pharmacokinetic endpoint study comprised of two age cohorts of patients with pJIA who were treated with SC abatacept (ClinicalTrials.gov identifier: NCT01844518; Supplementary Fig. 1). Patients aged 2–17 years were recruited from 50 sites in 12 countries: Argentina, Belgium, Brazil, France, Germany, Italy, Mexico, Peru, Russian Federation, Spain, South Africa, and the US. Patients received weekly SC abatacept for 4 months based on body-weight tier (10 to < 25 kg [50 mg dose], 25 to < 50 kg [87.5 mg dose], and ≥ 50 kg [125 mg dose]). After 4 months (primary endpoint), patients with ≥ 30% improvement in JIA-ACR criteria (JIA-ACR30) [22] could continue for an additional 20 months of treatment with open-label SC abatacept. Patients who did not achieve at least a JIA-ACR30 response at month 4 were given the option to continue SC abatacept for an additional 3 months and discontinue treatment if a JIA-ACR30 response was not attained by month 7.

Study details including SC weight-tiered dosing of abatacept have been reported previously [6, 7]. Briefly, patients were included if they met the International League of Associations for Rheumatology criteria for JIA in one of the following categories [23]: extended oligoarticular JIA, polyarticular rheumatoid factor (RF)-positive JIA, polyarticular RF-negative JIA, enthesitis-related JIA, psoriatic JIA, or systemic JIA (lacking systemic features for ≥ 6 months prior to enrollment). Patients were also required to have a history of ≥ 5 joints with active disease and active articular disease at baseline, defined as ≥ 2 active joints and ≥ 2 joints with limitation of motion at baseline. All patients were naive to treatment with abatacept but may have had an inadequate response or intolerance to ≥ 1 biologic or synthetic DMARD.

### Outcome measures

At each study visit in the phase 3 study [6], six JIA-ACR core set variables were measured: active joint count (AJC), number of joints with limitation of motion, Physician Global Assessment of disease activity (PhGA), Parent Global Assessment of patient overall well-being (PtGA), Childhood Health Assessment Questionnaire-Disability Index [24], and a laboratory marker of acute inflammation (CRP).

Considering the JIA-ACR core set variables, improvement from baseline was measured by JIA-ACR30/50/70/90/100 responses [22], or by achievement of inactive disease (JIA-ACR ID) [25]. Briefly, ID is defined as having no joints with active arthritis; no fever, rash, serositis, splenomegaly, or generalized lymphadenopathy attributable to JIA; no active uveitis as defined by the Standardization of Uveitis Nomenclature Working Group; ESR or CRP level within normal limits in the laboratory where tested or, if elevated, not attributable to JIA; PhGA score of best possible on the scale used; and duration of morning stiffness ≤ 15 min [25]. Further, the Juvenile Arthritis Disease Activity Score in 27 joints using CRP [JADAS27-CRP] was calculated, which considered the CRP value, AJC, PhGA, and PtGA. JADAS values of ≤ 3.8 were considered low disease activity (JADAS LDA) and values of *≤* 1, as inactive disease (JADAS-ID) [26–28].

### Biomarkers

Q2 Solutions (formerly Quintiles; Durham, NC, USA) performed high-sensitivity CRP testing. Assays of the S100 proteins were performed in the Multiplex Core within the Research Flow Cytometry Core of the Cincinnati Children’s Hospital Medical Center (CCHMC, Cincinnati, OH, USA). The S100 proteins were assayed in duplicates as previously published by CCHMC [14]. In brief, the Human S100A12/EN-RAGE ELISA Kit (Medical and Biological Laboratories Co., Japan) and Quantikine Human S100A8/S100A9 Heterodimer Immunoassay (R&D Systems, Minneapolis, MN, USA) utilized the quantitative sandwich enzyme immunoassay technique.

The laboratory technicians performing biomarker assays (S100A8/9, S100A12, and CRP) were blinded to the clinical and demographic data associated with a given serum sample. Normal clinical ranges are between 716 and 3004 ng/mL for S100A8/9 and between 32 and 385 ng/mL for S100A12 [14].

Based on the distribution of biomarker values in the current patient cohort, thresholds for each biomarker were as follows: S100A8/9: low, ≤ 2204.07 ng/mL; high normal to mildly elevated, > 2204.07 to ≤ 4870.47 ng/mL; elevated, > 4870.47 ng/mL; S100A12: low, ≤ 131.1 ng/mL; high normal to mildly elevated, > 131.1 to ≤ 298.1 ng/mL; elevated, > 298.1 ng/mL; and CRP: low, ≤ 0.1 mg/dL; high normal to mildly elevated, > 0.1 to ≤ 0.5 mg/dL; elevated, > 0.5 mg/dL. Biomarker levels were measured at each visit; baseline levels of biomarkers were measured just before starting SC abatacept treatment.

### Statistical analysis

This exploratory analysis evaluated whether baseline biomarker levels (S100A8/9, S100A12, and CRP) predict clinical response to abatacept treatment for pJIA. Thus, we assessed the association of baseline biomarker levels with treatment response (measured by JIA-ACR responses) and disease activity (measured by JADAS27-CRP). We also examined whether the combination of S100 protein levels and CRP could improve the predictive ability for strong clinical response to abatacept (JIA-ACR90/100/ID or JADAS-LDA/ID). Given the close association that we observed between levels of S100A8/9 and S100A12 (Spearman correlation coefficient: 0.91), combinations of these two biomarkers were not explored. Furthermore, we explored whether concurrent MTX therapy had any effect on the predictive value of the biomarkers studied.

Baseline demographics and disease characteristics were summarized as mean and standard deviation or median and range, as appropriate, for continuous numerical variables, and frequencies and percentages for nominal and categorical variables. Race was self-reported using a fixed set of categories. Spearman correlation coefficients were calculated to assess the association of baseline JADAS27-CRP with biomarker levels (S100A8/9, S100A12, CRP) measured at baseline. Values of Spearman correlation coefficients of < 0.2, 0.2 to < 0.4, 0.4 to < 0.6, 0.6 to < 0.8, and ≥ 0.8 were interpreted as unrelated, weakly, moderately, strongly, and almost perfectly associated, respectively [29]. Changes from baseline in biomarker levels by month 4 were also assessed for association with disease outcomes later in the study. Univariate logistic regression was used to estimate the ORs (95% CIs) of baseline biomarker values, individually or in selected combinations, to predict JIA outcomes post-baseline. We analyzed results up to month 21 with a focus on the results up to month 16. These exploratory post hoc analyses were not designed for formal inference/hypothesis testing. Missing values of efficacy response were imputed as non-responders.

## Results

### Patient population

**Fig. 1:**
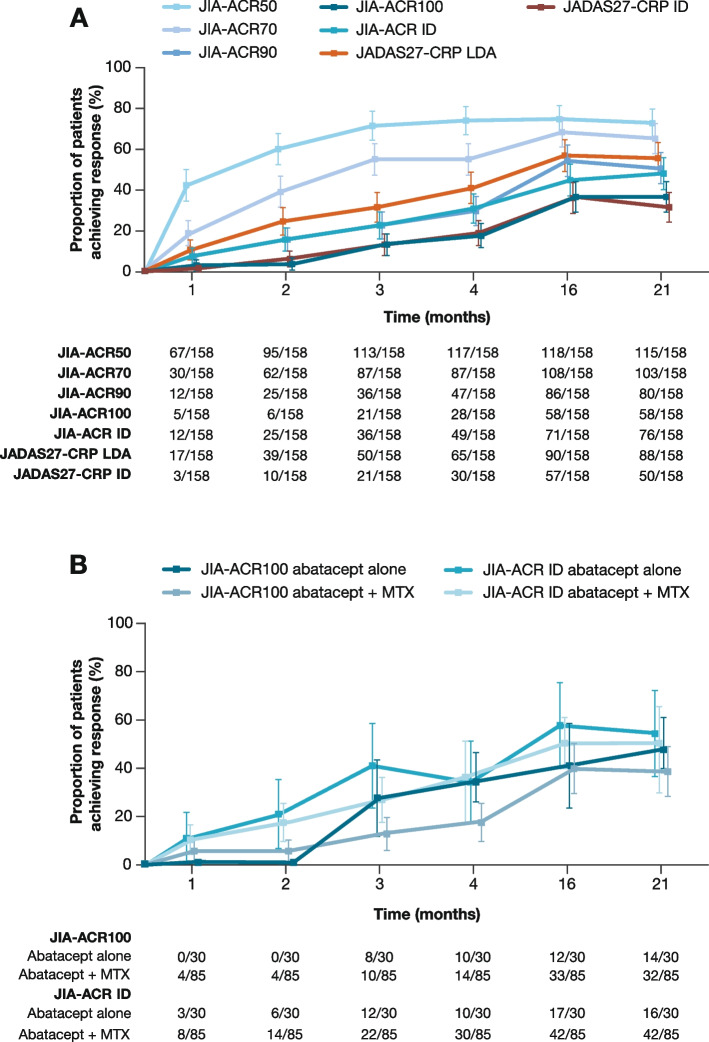
Clinical response to abatacept over 21 months for **A** the biomarker cohort* and **B** by background MTX use. Values are given as n/m, where n is the number of responders at the corresponding time point for each baseline category and m is the number of patients in each baseline category. *All treated patients with available baseline data for S100 proteins (S100A8/9 or S100A12). At months 4, 16, and 21, patients who were still in the study but had missing JADAS27-CRP values were considered as non-responders at those respective time points in the analysis (*n* = 3, 4, 2, respectively). Patients who were not in the study and had no available efficacy assessments at those respective time points and later time points were also considered as non-responders (*n* = 9, 26, 32, respectively). CRP = C-reactive protein; ID = inactive disease; JADAS27-CRP = Juvenile Arthritis Disease Activity Score in 27 joints using C-reactive protein; JIA-ACR50/70/90/100 = 50/70/90/100% improvement in juvenile idiopathic arthritis-American College of Rheumatology criteria; LDA = low disease activity; MTX = methotrexate

Serial samples from 158 of the 219 (72.1%) patients enrolled in the clinical trial were available for biomarker analysis. Demographics of patients included in this exploratory analysis were comparable with those of the overall clinical trial cohort (Table 1). Biomarker levels did not differ with age at baseline (Supplementary Table 1). Patients reported use of concomitant MTX and oral corticosteroids as background therapy starting at baseline (Table 1). No other concomitant medications were received. In the biomarker cohort, the trajectories of patients achieving JIA-ACR responses were similar to those achieving JADAS27-CRP responses (Fig. 1A). The proportion of patients receiving abatacept alone who achieved JIA-ACR100 and JIA-ACR ID over the 21-month study period was similar to that of patients receiving abatacept + MTX (Fig. 1B).

**Table 1:** Baseline demographics and disease characteristics

Characteristic	Biomarker cohort (*n* = 158)	Overall cohort (*N* = 219)
Age, years, mean ± SD	11.37 ± 4.0	10.58 ± 4.4
Female sex, n (%)	121 (76.6)	164 (74.9)
Race, ^a^ n (%)		
White	133 (84.2)	188 (85.8)
Black/African American	13 (8.2)	15 (6.8)
Other	12 (7.6)	16 (7.3)
Geographic region, n (%)		
North America	19 (12.0)	22 (10.0)
South America	52 (32.9)	64 (29.2)
Europe	73 (46.2)	114 (52.1)
Rest of the world	14 (8.9)	19 (8.7)
Disease duration, years, mean ± SD	2.56 ± 3.2	2.41 ± 3.0
pJIA categories, n (%)		
Polyarthritis RF negative	84 (53.2)	123 (56.2)
Polyarthritis RF positive Extended oligoarthritis	39 (24.7) 20 (12.7)	48 (21.9) 29 (13.2)
Systemic arthritis	4 (2.5)	5 (2.3)
Psoriatic arthritis	2 (1.3)	5 (2.3)
Enthesitis-related arthritis	4 (2.5)	4 (1.8)
Other^b^	5 (3.2)	5 (2.3)
JIA-ACR core set variables, mean ± SD		
Number of active joints	12.52 ± 8.0	11.76 ± 7.9
Number of joints with LOM	10.66 ± 8.2	10.30 ± 7.9
PtGA,^c^ mm	44.52 ± 25.5	44.13 ± 25.6
PhGA,^c^ mm	49.51 ± 20.7	48.22 ± 20.5
CHAQ-DI^d^	1.06 ± 0.7	1.01 ± 0.7
CRP, mg/dL^e^		
Median (range)	0.20 (0.1–20.5)	0.20 (0.1–21.1)
Mean ± SD	1.32 ± 2.7	1.24 ± 2.8
JADAS27-CRP, mean ± SD	19.83 ± 8.79	19.07 ± 8.8
MTX use, n (%)	124 (78.5)	172 (78.5)
MTX dose, mg/m^2^/week, mean ± SD	12.03 ± 4.3	12.28 ± 4.1
Oral corticosteroids, n (%)	46 (29.1)	65 (29.7)
S100A8/9 (ng/mL)^e^	3295.34	–
Median (range)	(544.3–160,193.3)	
Mean ± SD	9312.8 ± 20,890.3	
S100A12 (ng/mL)^e^		
Median (range)	176.5 (20.2–9485.6)	–
Mean ± SD	525.6 ± 1254.5	

*CHAQ-DI*  Childhood Health Assessment Questionnaire-Disability Index, *CRP*  C-reactive protein, *JADAS27-CRP*  Juvenile Arthritis Disease Activity Score in 27 joints using C-reactive protein, *JIA-ACR*  juvenile idiopathic arthritis-American College of Rheumatology criteria, *LOM*  limitation of motion, *MTX*  methotrexate, *PhGA*  Physician Global Assessment, *pJIA*  polyarticular-course juvenile idiopathic arthritis, *PtGA*  Parent Global Assessment of patient overall well-being, *RF*  rheumatoid factor, *SD*  standard deviation

^a^Race was self-reported. Patients chose from a fixed set of categories (White, Black or African American, Asian, American Indian or Alaska Native, Native Hawaiian or Pacific Islander, Other [specify – free text field]); the “Other” category included Turkish, North African, Mestizo, Mestiza, and mixed race

^b^“Other” category included persistent oligoarthritis and undifferentiated arthritis

^c^The PtGA and PhGA were each measured on a 0–100 mm visual analog scale, with higher values indicating greater disease activity or greater reduction in overall well-being, respectively

^d^CHAQ-DI measures physical function limitations on a 0–3 scale, across eight domains of disability components, with higher values indicating greater disability

^e^Normal clinical ranges for S100A8/9 are between 716 and 3004 ng/mL and for S100A12 are between 32 and 385 ng/mL [14]. Normal clinical levels for high-sensitivity CRP are ≤ 0.6 mg/dL

### Biomarker levels and their association with disease activity

At baseline, median serum levels were 3295.3 ng/mL for S100A8/9, 176.5 ng/mL for S100A12, and 0.20 mg/L for CRP. At month 4, the median serum level of S100A8/9 decreased to 2602 ng/mL, while median S100A12 level increased to 188.4 ng/mL and median CRP level remained unchanged at 0.20 mg/dL. At month 16, median serum levels of S100A8/9, S100A12, and CRP were 2449 ng/mL, 161.3 ng/mL, and 0.10 mg/dL, respectively.

At baseline, patients receiving MTX (*n* = 172) had numerically higher median S100A8/9 and S100A12 baseline levels than those receiving abatacept alone (*n* = 47; median [range] for S100A8/9: 3359 [544.3 − 160,193.3] vs. 2752 [612.9 − 76,786.7], *P* = 0.50; for S100A12: 178 [21.5 − 7810.3] vs. 161 [20.2 − 9485.6], *P* = 0.59). Likewise, baseline S100A8/9 levels were almost perfectly correlated with S100A12 and strongly correlated with CRP levels (*r* = 0.91 and *r* = 0.64, respectively; Supplementary Fig. 2A, 2B), while baseline CRP and S100A12 levels were moderately correlated with each other (*r* = 0.52; Supplementary Fig. 2C). At baseline, JADAS27-CRP values were poorly correlated with S100A8/9 levels (*r* = 0.24), S100A12 levels (*r* = 0.17), and CRP levels (*r* = 0.25) as shown in Supplementary Fig. 3A–C.

### Prediction of response to abatacept treatment

**Fig. 2:**
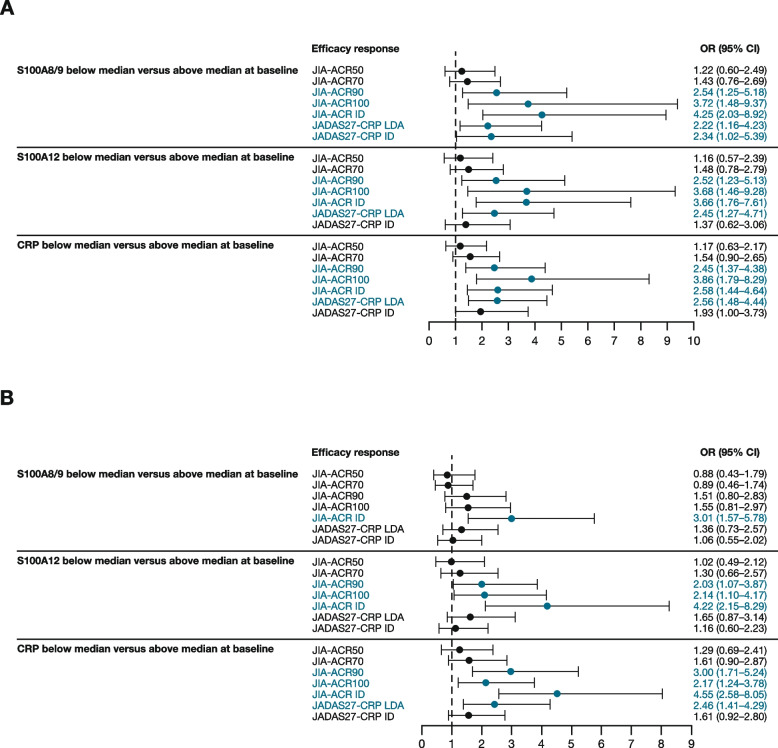
Prediction of response to abatacept treatment by baseline biomarker level at **A** month 4 and **B** month 16. CI = confidence interval; ID = inactive disease; JADAS27-CRP = Juvenile Arthritis Disease Activity Score in 27 joints using C-reactive protein; JIA-ACR50/70/90/100 = 50/70/90/100% improvement in juvenile idiopathic arthritis-American College of Rheumatology criteria; LDA = low disease activity; OR = odds ratio

Clinical outcomes by lower baseline biomarker levels. As shown in Fig. 2, while baseline levels of S100A8/9, S100A12, or CRP did not significantly predict JIA-ACR50 or JIA-ACR70 responses at months 4 or 16, they were significant predictors of greater clinical responses. For example, at month 4, patients with lower S100A8/9 or S100A12 levels at baseline had greater odds of achieving JIA-ACR90/100/ID and JADAS27-CRP LDA/ID (Supplementary Table 2; Fig. 2A). Similar observations were noted for CRP levels at months 4 and 16. At month 16, lower baseline CRP levels were associated with stronger clinical responses (Fig. 2B).

*Clinical outcomes by low baseline and 4-month biomarker levels.* The lower the S100A8/9 or S100A12 levels at baseline, the higher the likelihood of achieving JIA-ACR ID and JIA-ACR100 at months 4 and 16 (Supplementary Table 3). For example, low baseline S100A8/9 or S100A12 levels increased the odds of achieving JIA-ACR ID at month 4 by 9.1-fold or 5.8-fold, respectively; and at month 16 by 5.4-fold for both S100 proteins. Low CRP levels were also useful in predicting JIA-ACR100 and JIA-ACR ID responses (Supplementary Table 3).

When measured at month 4 instead of baseline, low levels of S100A8/9, S100A12, and CRP were also associated with higher odds of achieving JIA-ACR ID and JIA-ACR100 at months 16 and 21 (Supplementary Table 4).

*Early responses with combination of S100 proteins and CRP.* Combined with lower CRP, lower baseline S100A8/9 and S100A12 levels were associated with even higher odds of achieving JIA-ACR ID as early as month 3 (*P* = 0.0010 and *P* = 0.0092, respectively) compared with higher S100A8/9 or S100A12 levels alone (Supplementary Table 5). Lower baseline S100A8/9 levels increased the odds of achieving JIA-ACR100 at month 3 by 4.9-fold (95% CI: 1.56–15.24), while the combination of lower S100A8/9 plus lower CRP at baseline increased the likelihood of achieving JIA-ACR100 at month 3 by 6.9-fold (95% CI: 2.20–21.87). Lower baseline S100A12 levels increased the likelihood of achieving a JIA-ACR100 response at month 3 by 2.7-fold (95% CI: 0.97–7.28), whereas lower baseline S100A12 plus lower CRP increased the likelihood by 3.9-fold (95% CI: 1.40–10.67).

*Biomarker performance with or without concurrent MTX therapy.* Generally, at month 3, lower baseline S100A8/9 levels were associated with numerically higher odds of achieving JIA-ACR ID in patients receiving abatacept with MTX compared with patients receiving abatacept alone (Supplementary Table 6); a similar relationship was observed for S100A12. At months 4 and 16, lower S100A8/9 and S100A12 levels with or without lower CRP resulted in numerically higher odds of correctly predicting JIA-ACR ID response in patients receiving abatacept with MTX compared with patients receiving abatacept alone. Thus, use of either MTX only or both MTX and corticosteroids as background therapy during the study did not notably impact S100A8/9, S100A12, or CRP levels at any time point over 21 months (data not shown).

*Changes in biomarker levels from baseline as predictors of response to treatment.* Percent changes in biomarker levels are summarized in Supplementary Fig. 4. The mean CRP level decreased from baseline to month 4 and was maintained thereafter, whereas the mean S100A8/9 and S100A12 levels increased moderately up to month 21.

**Fig. 3:**
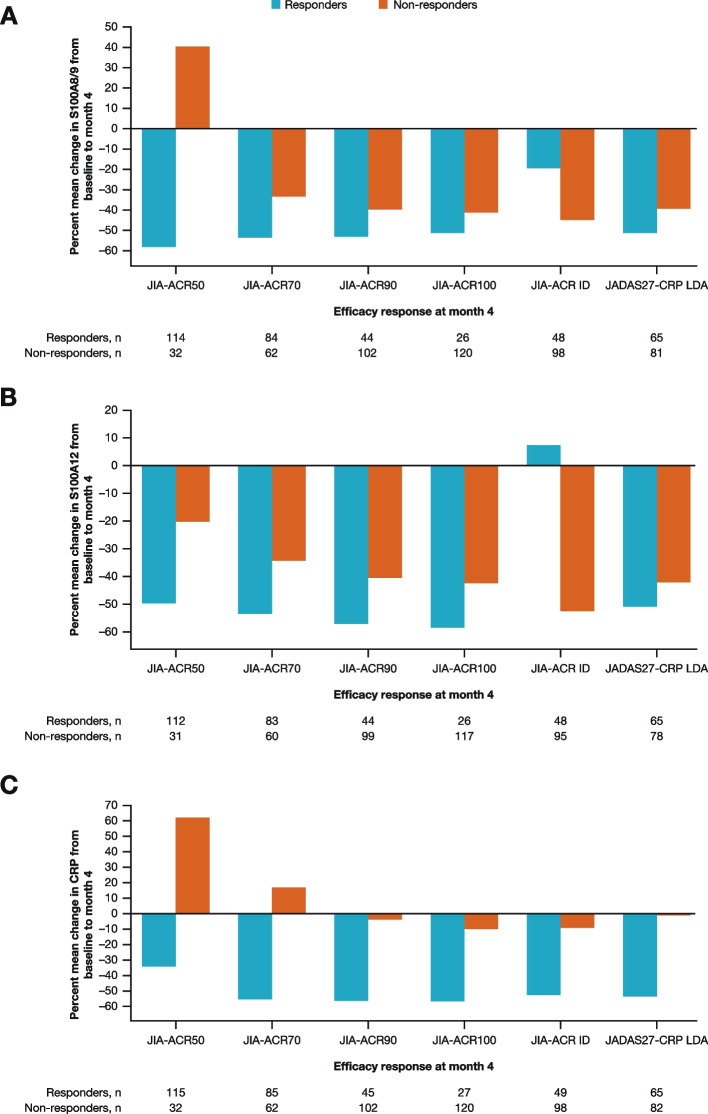
Percent mean change in biomarker levels of **A** S100A8/9, **B** S100A12, and **C** CRP from baseline to month 4 for responders versus non-responders across efficacy endpoints. CRP = C-reactive protein; ID = inactive disease; JADAS27-CRP = Juvenile Arthritis Disease Activity Score in 27 joints using C-reactive protein; JIA-ACR50/70/90/100 = 50/70/90/100% improvement in juvenile idiopathic arthritis-American College of Rheumatology criteria; LDA = low disease activity

Percentage mean changes in the serum S100 proteins and CRP levels from baseline to month 4 were differentiated between responders and non-responders (Fig. 3A–C). Overall, large decreases were observed in levels of S100A8/9 and S100A12 among responders from baseline to month 4 (Fig. 3A and B). The same held true for changes in CRP levels from baseline (Fig. 3C).

## Discussion

In this exploratory analysis of data from a phase 3 trial in patients with pJIA, lower baseline inflammatory biomarker levels predicted response to abatacept. Patients with low (compared with elevated) or lower than median baseline levels of S100A8/9, S100A12, and CRP had at least double the likelihood of achieving JIA-ACR ID or JIA-ACR100 after abatacept initiation. Decreases in S100 biomarker levels during the first 4 months of abatacept therapy may also predict longer-term response to abatacept in patients with pJIA. This seems congruent with prior research suggesting that S100A12 and CRP may help identify patients at risk of JIA flares, which could support decisions to taper, stop, or maintain treatment in different scenarios in JIA [30, 31].

Our findings are in line with previous studies that suggested an association between S100 protein levels and disease activity in patients with RA [12, 16, 17] and JIA [13, 14]. In these studies, high levels of S100 proteins predicted response in patients treated with adalimumab; in the current study, lower levels of S100 proteins predicted response in patients treated with abatacept. This difference may reflect the differing mechanisms of action of adalimumab and abatacept, but additional studies in other cohorts are needed to confirm this observation. Extracellular S100 proteins can act as damage-associated molecular patterns and activate innate immunity through toll-like receptors [14, 21, 32, 33]. Therefore, elevated levels of these biomarkers may reflect activation of innate immune responses. In contrast, abatacept controls dysregulated adaptive immune responses. We hypothesize that a subgroup of patients with pJIA exhibiting lower levels of S100 proteins may experience a profound response to abatacept and particularly benefit from this therapy.

Prior research showed that S100A12 and CRP may predict patients at risk of JIA flares, which may support treatment decisions in different populations of patients with JIA [30, 31]. An earlier controlled trial demonstrated that JIA flare risk after MTX withdrawal is independent of the duration of therapy or time in clinical remission [33]. A subsequent post hoc analysis revealed a similar flare risk between patients with low levels of S100A12 and CRP who were off medication and patients with JIA who continued MTX but had high levels of S100A12 and/or CRP [30, 31]; this finding suggested that S100 proteins, like CRP, are a good measure of inflammatory states in pJIA. A subsequent trial confirmed that the presence of both low CRP and low S100A12 levels in patients with JIA was associated with a decreased risk of JIA flare post DMARD withdrawal by 38% over 12 months (based on a hazard ratio of 0.62; *P* = 0.0455) compared with patients from a large registry in whom DMARD withdrawal occurred without consideration of biomarker levels [34].

In this study, MTX background therapy did not significantly influence the ability of S100 proteins to anticipate JIA improvement. Conversely, prior research found flare rates in patients with JIA treated with MTX to be more closely related to S100 levels than those in patients treated with biologic DMARDs (e.g. TNF inhibitors) [32]; additionally, high baseline S100A12 levels were found to predict improvement with MTX or TNF inhibitor therapy in patients with pJIA, with a decrease in S100A12 levels observed with treatment over time [21]. Taken together, the findings might suggest that interpretation of S100 proteins in JIA is dependent of the type of DMARD therapy considered, with lower levels being predictive of a substantial response to abatacept. Considering our recent study that showed that, in JIA, abatacept response was similar in patients receiving abatacept + MTX and those receiving abatacept monotherapy [35], the current data may support the hypothesis that S100 proteins are better biomarkers in patients for whom MTX therapy alone failed and who have therefore newly started combination therapy with abatacept + MTX. As our estimates were based on an exploratory analysis, future studies should assess the effect of abatacept on S100 biomarker levels and examine any potential contributions of the innate immune system to pJIA manifestations and the risk of flares.

Very high levels of S100A8/9 and S100A12 are reported in patients with systemic JIA, especially those with high disease activity [14, 36, 37]. In our study, S100A8/9 and S100A12 levels generally decreased more in patients with pJIA who responded to therapy by month 4 than those classified as non-responders. CRP levels were reduced in abatacept responders across various measurements of clinical outcomes. In contrast, increased CRP levels were observed in treatment non-responders.

Prior reports suggested that the combined assessment of CRP with S100A8/9 or S100A12 increases the precision with which JIA disease courses can be anticipated [30, 31]; our results confirm this observation. We noted that lower (below median) baseline CRP levels alone strongly predicted clinical response at 16 months (particularly JIA-ACR ID), supporting the value of CRP as a predictive biomarker of systemic inflammation. However, the combination of lower CRP and lower S100A8/9 or S100A12 levels increased the likelihood of high-level improvement (JIA-ACR100) 3 months after abatacept initiation; this combined assessment may have the potential to better predict disease-specific improvements in JIA.

The biomarkers included in this exploratory analysis only differentiated patients with a notable response to therapy but not those with less profound improvement of pJIA in response to abatacept (i.e. those with JIA-ACR30, 50, or 70 responses). We were unable to determine whether one S100 protein was a better predictor than the other because of the close correlation between S100A8/9 levels and S100A12 levels in patients with pJIA, the OR (95% CI) range overlap, and the differences in results for different clinical variables and time points. As such, we confirm that both S100A8/9 and S100A12 biomarker levels are useful to identify patients with pJIA who might respond well to abatacept and may help personalize JIA treatments as part of future treat-to-target strategies [3].

This study adds to the clinical evaluation of predictive biomarkers, but caution should be applied when interpreting these results, as these are post hoc analyses from an open-label study. Results after month 4 should be interpreted cautiously due to the study design, whereby non-responders could discontinue the study after month 4.

Limitations of this study include relatively small patient populations and the nature of the predictive analysis, whereby CRP was included as a component in the efficacy endpoints (i.e. ACR, JADAS); however, this was addressed by additional analyses evaluating whether including CRP as a biomarker enhanced the predictive value of the S100 biomarkers. Future analyses may explore this relationship using endpoints that exclude CRP as a confounding factor (e.g. clinical JADAS). The specific definitions of *low* and *lower* S100 protein levels in our study were selected to allow for logistical regression performance and presentation of odds ratios, which compared these levels to elevated or higher than median levels of S100 proteins, respectively, and may not be generalizable to datasets using other definitions. Finally, we were unable to confirm the relevance of our study findings for patients with certain racial backgrounds (e.g. Asian race).

In conclusion, lower levels of S100 proteins at baseline are predictive of more profound and sustained improvements in patients with pJIA treated with abatacept. The predictive value of S100 biomarkers for abatacept response was similar, irrespective of concurrent MTX use. Identifying patients with pJIA who may achieve early and greater clinical response with abatacept treatment could be a useful component of a precision-medicine approach in pJIA. Thus, levels of S100 proteins may be useful to predict pJIA courses in children for whom abatacept therapy is considered.

### Supplementary Information

Supplementary Material 1.

## Data Availability

The data generated or analyzed during this study are available from the corresponding author on reasonable request. Some data from this paper were presented at the 2021 EULAR European Congress of Rheumatology annual meeting (POS0076, Ruperto N, et al.).

## Abbreviations

ACR: American College of Rheumatology
AJC: Active joint count
CCHMC: Cincinnati Children’s Hospital Medical Center
CHAQ-DI: Childhood Health Assessment Questionnaire-Disability Index
CI: Confidence interval
C_minss_: Minimum abatacept concentration
CRP: C-reactive protein
DMARDs: Disease-modifying antirheumatic drugs
ESR: Erythrocyte sedimentation rate
ID: Inactive disease
JADAS: Juvenile Arthritis Disease Activity Score
JADAS27-CRP: Juvenile Arthritis Disease Activity Score in 27 joints using C-reactive protein
JIA: Juvenile idiopathic arthritis
JIA-ACR50/70/90/100: 50/70/90/100% improvement in juvenile idiopathic arthritis-American College of Rheumatology criteria
LDA: Low disease activity
LOM: Limitation of motion
MTX: Methotrexate
OR: Odds ratio
PhGA: Physician Global Assessment of disease activity
pJIA: Polyarticular-course juvenile idiopathic arthritis
PtGA: Parent Global Assessment of patient overall well-being
RA: Rheumatoid arthritis
RF: Rheumatoid factor
SC: Subcutaneous
SD: Standard deviation
TNF: Tumor necrosis factor
