# Correction: S100 proteins as potential predictive biomarkers of abatacept response in polyarticular juvenile idiopathic arthritis

**DOI:** 10.1186/s13075-024-03385-8

**Published:** 2024-08-31

**Authors:** Hermine I Brunner, Grant S Schulert, Alyssa Sproles, Sherry Thornton, Gabriel Vega Cornejo, Jordi Antón, Ruben Cuttica, Michael Henrickson, Ivan Foeldvari, Daniel J Kingsbury, Margarita Askelson, Jinqi Liu, Sumanta Mukherjee, Robert L Wong, Daniel J Lovell, Alberto Martini, Nicolino Ruperto, Alexei A Grom, on behalf of the Pediatric Rheumatology Collaborative Study Group (PRCSG) and the Paediatric Rheumatology International Trials Organisation (PRINTO)

**Affiliations:** 1 Division of Rheumatology, Department of Pediatrics, Cincinnati Children’s Hospital Medical Center, University of Cincinnati College of Medicine, Cincinnati, OH USA; 2 Hospital México Americano, Guadalajara, CREA Mexico; 3 Pediatric Rheumatology Department, Hospital Sant Joan de Deu, Universitat de Barcelona, Barcelona, Spain; 4 Ruben Cuttica MD, Pediatric Rheumatology, Hospital General de Ninos Pedro de Elizalde, Buenos Aires, Argentina; 5 Hamburg Centre for Pediatric and Adolescent Rheumatology, Schon Klinik Hamburg Eilbek, Hamburg, Germany; 6 Division of Rheumatology, Randall Children’s Hospital at Legacy Emanuel, Portland, OR USA; 7 Global Biometric Sciences, Bristol Myers Squibb, Princeton, NJ USA; 8 Translational Medicine, Bristol Myers Squibb, Princeton, NJ USA; 9 Bristol Myers Squibb, Immunology and Fibrosis, Princeton, NJ USA; 10 Dipartimento di Neuroscienze, Riabilitazione, Oftalmologia, Genetica e Scienze Materno-Infantili (DiNOGMI), Universita degli Studi di Genova, Genoa, Italy; 11 IRCCS Istituto Giannina Gaslini, Gaslini Trial Centre/Servizio di Sperimentazioni Cliniche Pediatriche, PRINTO, Genoa, Italy

**Correction: Arthritis Res Ther 26**,** 125 (2024)**

10.1186/s13075-024-03347-0

Following publication of the original article [1], the authors reported an error to Supplementary Material 2. Supplementary Material 2 was removed as the file was only for the reviewers’ reference and not meant to be published.

The original article [1] has been updated.
