# Supplementary material for: S100 proteins as potential predictive biomarkers of abatacept response in polyarticular juvenile idiopathic arthritis
Source: Arthritis Res Ther. 2024 Jun 25;26:125. doi: 10.1186/s13075-024-03347-0 (PMC11197242; doi:10.1186/s13075-024-03347-0)
Supplement: Supplementary file 1 — Supplementary Material 1. [file 13075_2024_3347_MOESM1_ESM.pdf]

S100 Proteins as Potential Predictive Biomarkers of Abatacept Response in Polyarticular Juvenile Idiopathic Arthritis. Brunner HI, et al.

Supplementary Methods. Protocol amendments after study commencement

After study commencement, a few changes were made to the study protocol, including revisions to the pregnancy test requirements and additional minor changes. At a few study sites, changes to HIV testing at screening were incorporated and enrollment was restricted to children and adolescents 6–17 years of age at screening. Some amendments were made to the inclusion and exclusion criteria, including clarifications for cases of drug intolerance, randomization limits for each cohort, language regarding mandatory contraception requirements, and removal of psoriatic juvenile idiopathic arthritis (JIA) and enthesitis-related JIA as exclusion criteria. Finally, clarification was added to note that the 7 days post-dose follow-up visit was performed as the early termination/final study completion visit and objectives/endpoints were updated to align with the current regulatory view on long-term safety analysis.

Supplementary Figure 1. Study design: single-arm open-label phase 3 study of SC abatacept.

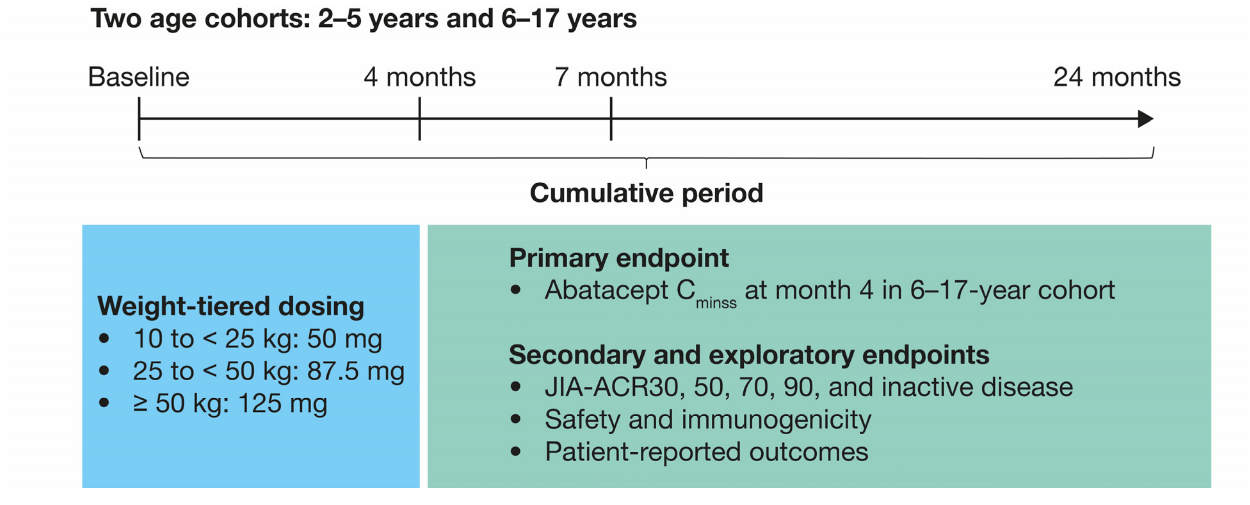

Key eligibility criteria: 2–17 years of age, male or female sex, pJIA, active articular disease (≥ 2 active joints and ≥ 2 joints with LOM), insufficient response/intolerance to ≥ 1 non-biologic or biologic DMARD

C_{minss} = minimum abatacept concentration; DMARD = disease-modifying antirheumatic drug; JIA-ACR30/50/70/90 = 30/50/70/90% improvement in juvenile idiopathic arthritis-American College of Rheumatology criteria; LOM = limitation of motion; pJIA = polyarticular-course juvenile idiopathic arthritis; SC = subcutaneous.

Supplementary Figure 2. Correlation between (A) S100A8/9 and S100A12, (B) S100A8/9 and CRP, and (C) S100A12 and CRP levels at baseline in the biomarker cohort (N = 158).

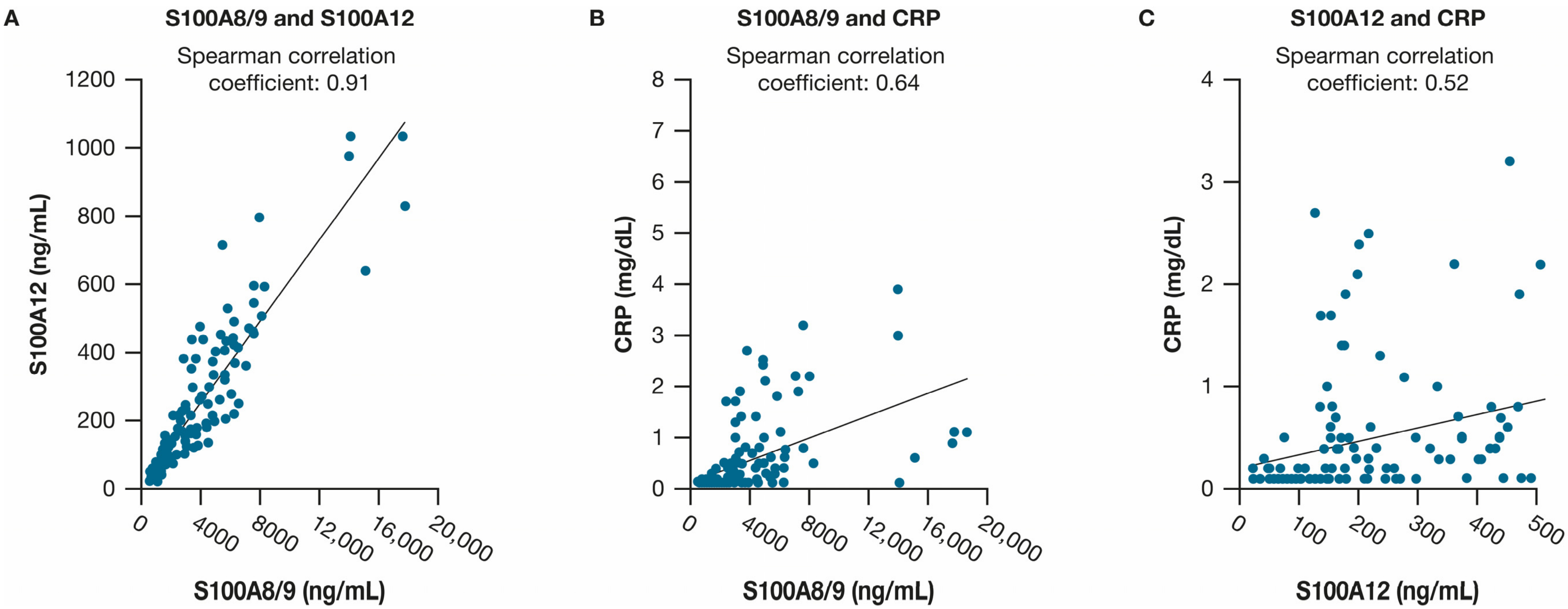

Correlation coefficients of 0.4 to ≤ 0.6 were considered as moderately correlated, > 0.6 to ≤ 0.8 as strongly correlated, and > 0.8 as almost perfect agreement.

The number of observations per plot are unavailable. Extreme outliers were excluded from the analysis; an outlier was considered extreme if it sat beyond the lower outer fence: Q1 - 3*(Q3–Q1) or the upper outer fence: Q3 + 3*(Q3–Q1).

CRP = C-reactive protein.

S100 Proteins as Potential Predictive Biomarkers of Abatacept Response in Polyarticular Juvenile Idiopathic Arthritis. Brunner HI, et al.

Supplementary Figure 3. Correlation between baseline (A) S100A12, (B) S100A8/9, and (C) CRP levels and baseline disease activity measured by JADAS27-CRP.

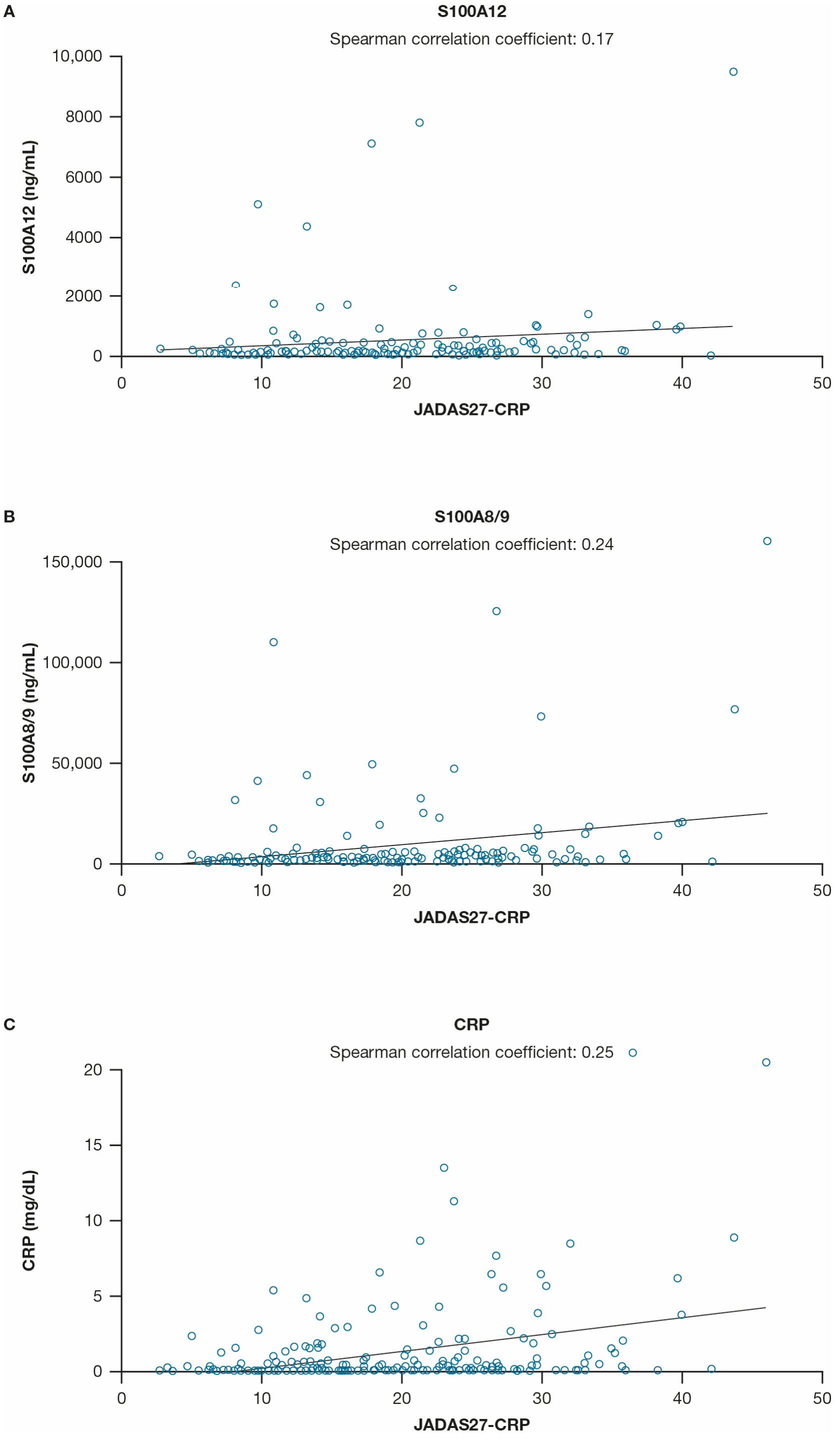

CRP = C-reactive protein; JADAS27-CRP = Juvenile Arthritis Disease Activity Score in 27 joints using C-reactive protein.

S100 Proteins as Potential Predictive Biomarkers of Abatacept Response in Polyarticular Juvenile Idiopathic Arthritis. Brunner HI, et al.

Supplementary Figure 4. Change in biomarker levels of S100A8/9, S100A12, and CRP from baseline to 3, 4, 16, and 21 months.

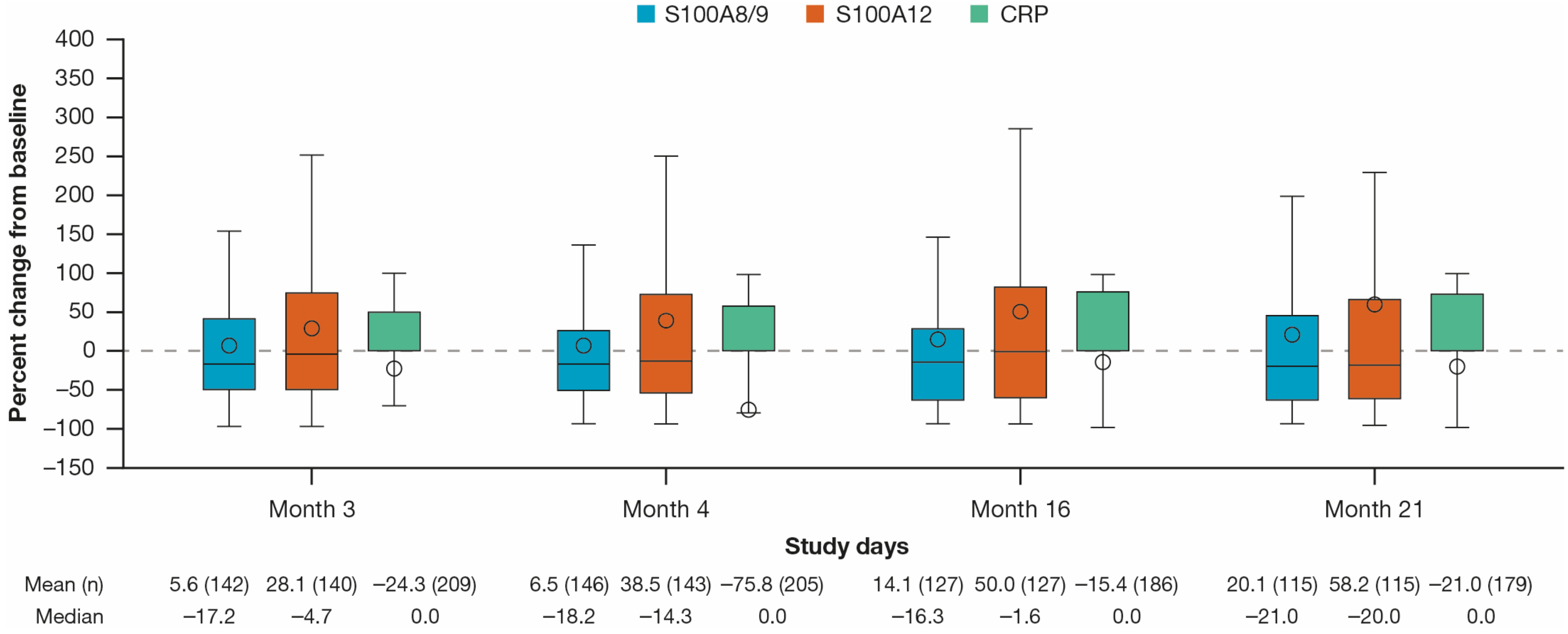

Lines within each boxplot indicate median value and circles indicate mean values. Extreme outliers are not displayed in the figure. Baseline is day 1 of the study. CRP = C-reactive protein.

Supplementary Table 1. Biomarker levels in the biomarker cohort (n = 158) according to the two age groups

	Patient age group	
	**2–5 years (n = 19)**	**6–17 years (n = 139)**
**CRP level, mg/dL**		
Mean (SD)	1.93 (3.2)	1.23 (2.6)
Median (range)	0.20 (0.1–11.3)	0.30 (0.1–20.5)
**S100A8/9, ng/mL**		
Mean (SD)	15,784.84 (29,521.0)	8428.15 (19,397.4)
Median (range)	2999.27 (634.9–109,893.3)	3317.80 (544.3–160,193.3)
**S100A12, ng/mL**		
Mean (SD)	436.46 (646.0)	537.32 (1314.9)
Median (range)	154.64 (30.2–2303.3)	177.60 (20.2–9485.6)

CRP = C-reactive protein; SD = standard deviation.

Supplementary Table 2. Odds ratios (95% CI) of baseline biomarker levels (relative to median) to JIA-ACR ID response in biomarker cohort.

	**Biomarker cohort (n = 158)**	
	**OR (95% CI)**	***P* value***
Month 3		
**Lower S100A8/9**	3.81 (1.65–8.80)	0.0018
**Lower S100A8/9 + lower CRP**	4.79 (2.03–11.31)	0.0004
**Lower S100A12**	2.26 (1.03–4.95)	0.0415
**Lower S100A12 + lower CRP**	3.06 (1.36–6.85)	0.0067
Month 4		
**Lower S100A8/9**	3.99 (1.90–8.40)	0.0003
**Lower S100A8/9 + lower CRP**	4.62 (2.13–10.00)	0.0001
**Lower S100A12**	3.43 (1.65–7.15)	0.0010
**Lower S100A12 + lower CRP**	3.82 (1.77–8.23)	0.0006
Month 16		
**Lower S100A8/9**	2.96 (1.53–5.73)	0.0013
**Lower S100A8/9 + lower CRP**	3.51 (1.75–7.03)	0.0004
**Lower S100A12**	4.18 (2.11–8.29)	< 0.0001
**Lower S100A12 + lower CRP**	5.13 (2.47–10.62)	< 0.0001

Lower S100A8/9: ≤ 3295 ng/mL; lower S100A12: ≤ 176 ng/mL; lower CRP: ≤ 0.2 mg/dL.

Missing values were imputed as non-responders of JIA-ACR ID.

*Combination of baseline S1008/9 and S100A12 levels versus above-median S100A12 levels.

CI = confidence interval; CRP = C-reactive protein; JIA-ACR ID = juvenile idiopathic arthritis-American College of Rheumatology criteria for inactive disease; OR = odds ratio.

Supplementary Table 3. Response to abatacept at months 4 and 16 by baseline biomarker levels relative to median.

Response	Visit	Biomarker	Baseline levels	n/m (%)	OR (95% CI)	*P* value
**JIA-ACR ID**						
	Month 4	S100A8/9	Low	26/53 (49.1)	9.05 (3.11–26.33)	0.0003
			High normal to mildly elevated	18/53 (34.0)	4.83 (1.64–14.28)	0.2333
			Elevated	5/52 (9.6)		
		S100A12	Low	25/52 (48.1)	5.82 (2.22–15.28)	0.0013
			High normal to mildly elevated	17/52 (32.7)	3.05 (1.14–8.18)	0.5406
			Elevated	7/51 (13.7)		
		CRP	Low	42/93 (45.2)	5.11 (2.33–11.17)	0.0041
			High normal to mildly elevated	23/54 (42.6)	4.60 (1.95–10.85)	0.0366
			Elevated	10/72 (13.9)		
	Month 16	S100A8/9	Low	34/53 (64.2)	5.37 (2.31–12.46)	0.0006
			High normal to mildly elevated	24/53 (45.3)	2.48 (1.08–5.69)	0.8434
			Elevated	13/52 (25.0)		
		S100A12	Low	35/52 (67.3)	5.44 (2.34–12.67)	0.0002
			High normal to mildly elevated	22/52 (42.3)	1.94 (0.85–4.42)	0.6006
			Elevated	14/51 (27.5)		
		CRP	Low	65/93 (69.9)	6.96 (3.48–13.93)	< 0.0001
			High normal to mildly elevated	27/54 (50.0)	3.00 (1.41–6.38)	0.6929
			Elevated	18/72 (25.0)		
**JIA-ACR90**						
	Month 4	S100A8/9	Low	22/53 (41.5)	3.90 (1.54–9.90)	0.0166
			High normal to mildly elevated	17/53 (32.1)	2.60 (1.01–6.71)	0.4694
			Elevated	8/52 (15.4)		
		S100A12	Low	22/52 (42.3)	3.01 (1.24–7.27)	0.0204
			High normal to mildly elevated	15/52 (28.8)	1.66 (0.67–4.15)	0.9114
			Elevated	10/51 (19.6)		
		CRP	Low	41/93 (44.1)	3.00 (1.49–6.04)	0.0168
			High normal to mildly elevated	20/54 (37.0)	2.24 (1.01–4.94)	0.4435
			Elevated	15/72 (20.8)		
	Month 16	S100A8/9	Low	34/53 (64.2)	2.86 (1.30–6.32)	0.0827
			High normal to mildly elevated	32/53 (60.4)	2.44 (1.11–5.34)	0.2911
			Elevated	20/52 (38.5)		

Response	Visit	Biomarker	Baseline levels	n/m (%)	OR (95% CI)	*P* value
		S100A12	Low	36/52 (69.2)	2.74 (1.22–6.14)	0.0155
			High normal to mildly elevated	27/52 (51.9)	1.32 (0.61–2.85)	0.5056
			Elevated	23/51 (45.1)		
		CRP	Low	67/93 (72.0)	4.05 (2.10–7.80)	0.0035
			High normal to mildly elevated	35/54 (64.8)	2.90 (1.39–6.02)	0.2711
			Elevated	28/72 (38.9)		
**JIA-ACR100**						
	Month 4	S100A8/9	Low	18/53 (34.0)	8.40 (2.30–30.73)	0.0003
			High normal to mildly elevated	7/53 (13.2)	2.49 (0.61–10.19)	0.7692
			Elevated	3/52 (5.8)		
		S100A12	Low	18/52 (34.6)	6.22 (1.93–20.03)	0.0003
			High normal to mildly elevated	6/52 (11.5)	1.53 (0.41–5.79)	0.3551
			Elevated	4/51 (7.8)		
		CRP	Low	26/93 (28.0)	6.60 (2.18–19.93)	0.0087
			High normal to mildly elevated	14/54 (25.9)	5.95 (1.83–19.32)	0.0452
			Elevated	4/72 (5.6)		
	Month 16	S100A8/9	Low	26/53 (49.1)	2.89 (1.26–6.61)	0.0214
			High normal to mildly elevated	19/53 (35.8)	1.68 (0.72–3.89)	0.9692
			Elevated	13/52 (25.0)		
		S100A12	Low	26/52 (50.0)	2.92 (1.27–6.72)	0.0208
			High normal to mildly elevated	19/52 (36.5)	1.68 (0.72–3.92)	0.9649
			Elevated	13/51 (25.5)		
		CRP	Low	46/93 (49.5)	2.94 (1.50–5.74)	0.0170
			High normal to mildly elevated	23/54 (42.6)	2.23 (1.04–4.75)	0.4196
			Elevated	18/72 (25.0)		
**JADAS27-CRP LDA**						
	Month 4	S100A8/9	Low	29/53 (54.7)	4.50 (1.91–10.62)	0.0102
			High normal to mildly elevated	25/53 (47.2)	3.33 (1.41–7.84)	0.2006
			Elevated	11/52 (21.2)		
		S100A12	Low	26/52 (50.0)	2.92 (1.27–6.72)	0.1246
			High normal to mildly elevated	26/52 (50.0)	2.92 (1.27–6.72)	0.1246
			Elevated	13/51 (25.5)		
		CRP	Low	53/93 (57.0)	3.22 (1.67–6.18)	0.0100
			High normal to mildly elevated	27/54 (50.0)	2.43 (1.16–5.07)	0.3425

Response	Visit	Biomarker	Baseline levels	n/m (%)	OR (95% CI)	*P* value
			Elevated	21/72 (29.2)		
	Month 16	S100A8/9	Low	34/53 (64.2)	1.79 (0.82–3.91)	0.1957
			High normal to mildly elevated	30/53 (56.6)	1.30 (0.61–2.81)	0.9410
			Elevated	26/52 (50.0)		
		S100A12	Low	31/52 (59.6)	1.21 (0.56–2.65)	0.7805
			High normal to mildly elevated	31/52 (59.6)	1.21 (0.56–2.65)	0.7805
			Elevated	28/51 (54.9)		
		CRP	Low	68/93 (73.1)	3.40 (1.77–6.53)	0.0029
			High normal to mildly elevated	33/54 (61.1)	1.96 (0.96–4.03)	0.8457
			Elevated	32/72 (44.4)		
**JADAS27-CRP ID**						
	Month 4	S100A8/9	Low	16/53 (30.2)	4.07 (1.36–12.12)	0.0109
			High normal to mildly elevated	9/53 (17.0)	1.92 (0.60–6.18)	0.9178
			Elevated	5/52 (9.6)		
		S100A12	Low	12/52 (23.1)	2.25 (0.77–6.55)	0.3427
			High normal to mildly elevated	12/52 (23.1)	2.25 (0.77–6.55)	0.3427
			Elevated	6/51 (11.8)		
		CRP	Low	25/93 (26.9)	2.94 (1.24–6.99)	0.1589
			High normal to mildly elevated	16/54 (29.6)	3.37 (1.32–8.61)	0.0689
			Elevated	8/72 (11.1)		
	Month 16	S100A8/9	Low	19/53 (35.8)	1.06 (0.47–2.35)	0.9692
			High normal to mildly elevated	20/53 (37.7)	1.15 (0.52–2.54)	0.7568
			Elevated	18/52 (34.6)		
		S100A12	Low	19/52 (36.5)	0.97 (0.44–2.16)	0.9652
			High normal to mildly elevated	19/52 (36.5)	0.97 (0.44–2.16)	0.9652
			Elevated	19/51 (37.3)		
		CRP	Low	39/93 (41.9)	2.17 (1.10–4.25)	0.3599
			High normal to mildly elevated	26/54 (48.1)	2.79 (1.31–5.93)	0.0476
			Elevated	18/72 (25.0)		

S100A8/9 levels: low, ≤ 2204.07 ng/mL; high normal to mildly elevated, > 2204.07 to ≤ 4870.47 ng/mL; elevated, > 4870.47 ng/mL. S100A12 levels: low, ≤ 131.1 ng/mL; high normal to mildly elevated, > 131.1 to ≤ 298.1 ng/mL; elevated, > 298.1 ng/mL. CRP levels: low, ≤ 0.1 mg/dL; high normal to mildly elevated, > 0.1 to ≤ 0.5 mg/dL; elevated, > 0.5 mg/dL. N is the number of responders at the

corresponding time point for each baseline category; m is the number of patients in each baseline category. Baseline is day 1 of the study. ORs, 95% CIs, and *P* values are based on univariate logistic regression model with baseline S100A8/9, S100A12, or CRP categories as single covariates. The elevated category is the reference group for the ORs. Missing values of efficacy response are imputed as non-responders.

CI = confidence interval; CRP = C-reactive protein; ID = inactive disease; JADAS27-CRP = Juvenile Arthritis Disease Activity Score in 27 joints using C-reactive protein; JIA-ACR90/100 = 90/100% improvement in juvenile idiopathic arthritis-American College of Rheumatology criteria; LDA = low disease activity; OR = odds ratio.

Supplementary Table 4. JIA-ACR ID response to abatacept at months 16 and 21 by biomarker levels relative to median.

Visit	Biomarker	Biomarker levels	n/m (%)	OR (95% CI)	*P* value
**Per month 4 biomarker levels***					
Month 16	S100A8/9	Low	48/68 (70.6)	6.06 (2.88–12.77)	0.0002
		High normal to mildly elevated	39/68 (57.4)	3.40 (1.66–7.00)	0.2993
		Elevated	19/67 (28.4)		
	S100A12	Low	45/67 (67.2)	4.71 (2.26–9.78)	0.0037
		High normal to mildly elevated	41/68 (60.3)	3.49 (1.71–7.14)	0.1247
		Elevated	20/66 (30.3)		
	CRP	Low	76/110 (69.1)	6.86 (3.37–13.93)	< 0.0001
		High normal to mildly elevated	17/34 (50.0)	3.07 (1.26–7.47)	0.6835
		Elevated	15/61 (24.6)		
Month 21	S100A8/9	Low	45/68 (66.2)	3.29 (1.62–6.65)	0.0119
		High normal to mildly elevated	39/68 (57.4)	2.26 (1.13–4.50)	0.4692
		Elevated	25/67 (37.3)		
	S100A12	Low	41/67 (61.2)	2.59 (1.29–5.20)	0.1608
		High normal to mildly elevated	43/68 (63.2)	2.82 (1.40–5.68)	0.0684
		Elevated	25/66 (37.9)		
	CRP	Low	73/110 (66.4)	4.04 (2.08–7.86)	0.0005
		High normal to mildly elevated	17/34 (50.0)	2.05 (0.87–4.84)	0.9601
		Elevated	20/61 (32.8)		
**Per baseline biomarker levels†**					
Month 16	S100A8/9	Low	34/53 (64.2)	5.37 (2.31–12.46)	0.0006
		High normal to mildly elevated	24/53 (45.3)	2.48 (1.08–5.69)	0.8434
		Elevated	13/52 (25.0)		
	S100A12	Low	35/52 (67.3)	5.44 (2.34–12.67)	0.0002
		High normal to mildly elevated	22/52 (42.3)	1.94 (0.85–4.42)	0.6006
		Elevated	14/51 (27.5)		
	CRP	Low	65/93 (69.9)	6.96 (3.48–13.93)	< 0.0001
		High normal to mildly elevated	27/54 (50.0)	3.00 (1.41–6.38)	0.6929
		Elevated	18/72 (25.0)		
Month 21	S100A8/9	Low	29/53 (54.7)	1.78 (0.82–3.87)	0.2349
		High normal to mildly elevated	26/53 (49.1)	1.42 (0.66–3.08)	0.8538

S100 Proteins as Potential Predictive Biomarkers of Abatacept Response in Polyarticular Juvenile Idiopathic Arthritis. Brunner HI, et al.

Visit	Biomarker	Biomarker levels	n/m (%)	OR (95% CI)	*P* value
		Elevated	21/52 (40.4)		
	S100A12	Low	30/52 (57.7)	1.80 (0.82–3.93)	0.1264
		High normal to mildly elevated	24/52 (46.2)	1.13 (0.52–2.46)	0.6170
		Elevated	22/51 (43.1)		
	CRP	Low	59/93 (63.4)	4.84 (2.47–9.49)	0.0038
		High normal to mildly elevated	33/54 (61.1)	4.38 (2.06–9.35)	0.0354
		Elevated	19/72 (26.4)		

*S100A8/9 levels: low, ≤ 1991.27 ng/mL; high normal to mildly elevated, > 1991.27 to ≤ 3663.8 ng/mL; elevated, > 3663.8 ng/mL. S100A12 levels: low, ≤ 130 ng/mL; high normal to mildly elevated, > 130 to ≤ 253 ng/mL; elevated, > 253 ng/mL. CRP levels: low, ≤ 0.1 mg/dL; high normal to mildly elevated, > 0.1 to ≤ 0.4 mg/dL; elevated, > 0.4 mg/dL.

†S100A8/9 levels: low, ≤ 2204.07 ng/mL; high normal to mildly elevated, > 2204.07 to ≤ 4870.47 ng/mL; elevated, > 4870.47 ng/mL. S100A12 levels: low, ≤ 131.1 ng/mL; high normal to mildly elevated, > 131.1 ng/mL to ≤ 298.1 ng/mL; elevated, > 298.1 ng/mL. CRP levels: low, ≤ 0.1 mg/dL; high normal to mildly elevated, > 0.1 to ≤ 0.5 mg/dL; elevated, > 0.5 mg/dL.

N is the number of responders at the corresponding time point for each category; m is the number of patients in each category. Baseline is day 1 of the study. ORs, 95% CIs, and *P* values are based on univariate logistic regression model with baseline S100A8/9 or S100A12 or CRP categories as single covariate. The elevated category is the reference group for the ORs. Missing values of efficacy response are imputed as non-responders.

CI = confidence interval; CRP = C-reactive protein; JIA-ACR ID = juvenile idiopathic arthritis-American College of Rheumatology criteria for inactive disease; OR = odds ratio.

Supplementary Table 5. JIA-ACR100 response with biomarkers and their combinations relative to median.

Biomarker	Visit	Baseline factor	n/m (%)	OR (95% CI)	*P* value
**S100A8/9 without CRP**					
	Month 1	Lower S100A8/9	4/79 (5.1)	3.95 (0.43–36.15)	0.2244
		Higher S100A8/9	1/75 (1.3)		
	Month 2	Lower S100A8/9	5/79 (6.3)	5.00 (0.57–43.84)	0.1462
		Higher S100A8/9	1/75 (1.3)		
	Month 3	Lower S100A8/9	17/79 (21.5)	4.87 (1.56–15.24)	0.0066
		Higher S100A8/9	4/75 (5.3)		
	Month 4	Lower S100A8/9	21/79 (26.6)	3.52 (1.40–8.86)	0.0077
		Higher S100A8/9	7/75 (9.3)		
	Month 16	Lower S100A8/9	33/79 (41.8)	1.52 (0.79–2.95)	0.2104
		Higher S100A8/9	24/75 (32.0)		
	Month 21	Lower S100A8/9	33/79 (41.8)	1.52 (0.79–2.95)	0.2104
		Higher S100A8/9	24/75 (32.0)		
**S100A8/9 with CRP**					
	Month 1	Lower S100A8/9 + lower CRP	4/63 (6.3)	5.29 (0.58–48.56)	0.1410
		Higher S100A8/9	1/79 (1.3)		
	Month 2	Lower S100A8/9 + lower CRP	5/63 (7.9)	6.72 (0.77–59.11)	0.0857
		Higher S100A8/9	1/79 (1.3)		
	Month 3	Lower S100A8/9 + lower CRP	17/63 (27.0)	6.93 (2.20–21.87)	0.0010
		Higher S100A8/9	4/79 (5.1)		
	Month 4	Lower S100A8/9 + lower CRP	21/63 (33.3)	5.14 (2.02–13.11)	0.0006
		Higher S100A8/9	7/79 (8.9)		
	Month 16	Lower S100A8/9 + lower CRP	30/63 (47.6)	1.96 (0.99–3.90)	0.0535
		Higher S100A8/9	25/79 (31.6)		
	Month 21	Lower S100A8/9 + lower CRP	28/63 (44.4)	1.73 (0.87–3.43)	0.1186
		Higher S100A8/9	25/79 (31.6)		

Biomarker	Visit	Baseline factor	n/m (%)	OR (95% CI)	*P* value
**S100A12 without CRP**					
	Month 1	Lower S100A12	4/78 (5.1)	3.89 (0.43–35.66)	0.2292
		Higher S100A12	1/73 (1.4)		
	Month 2	Lower S100A12	5/78 (6.4)	4.93 (0.56–43.26)	0.1498
		Higher S100A12	1/73 (1.4)		
	Month 3	Lower S100A12	15/78 (19.2)	2.66 (0.97–7.28)	0.0571
		Higher S100A12	6/73 (8.2)		
	Month 4	Lower S100A12	21/78 (26.9)	3.47 (1.38–8.77)	0.0084
		Higher S100A12	7/73 (9.6)		
	Month 16	Lower S100A12	36/78 (46.2)	2.12 (1.08–4.17)	0.0288
		Higher S100A12	21/73 (28.8)		
	Month 21	Lower S100A12	35/78 (44.9)	1.89 (0.97–3.69)	0.0633
		Higher S100A12	22/73 (30.1)		
**S100A12 with CRP**					
	Month 1	Lower S100A12 + lower CRP	4/61 (6.6)	5.33 (0.58–49.01)	0.1391
		Higher S100A12	1/77 (1.3)		
	Month 2	Lower S100A12 + lower CRP	5/61 (8.2)	6.79 (0.77–59.70)	0.0844
		Higher S100A12	1/77 (1.3)		
	Month 3	Lower S100A12 + lower CRP	15/61 (24.6)	3.86 (1.40–10.67)	0.0092
		Higher S100A12	6/77 (7.8)		
	Month 4	Lower S100A12 + lower CRP	20/61 (32.8)	4.88 (1.90–12.52)	0.0010
		Higher S100A12	7/77 (9.1)		
	Month 16	Lower S100A12 + lower CRP	30/61 (49.2)	2.42 (1.20–4.89)	0.0140
		Higher S100A12	22/77 (28.6)		
	Month 21	Lower S100A12 + lower CRP	29/61 (47.5)	2.13 (1.06–4.29)	0.0346
		Higher S100A12	23/77 (29.9)		
**CRP**					
	Month 3	Lower CRP	26/116 (22.4)	4.67 (1.84–11.87)	0.0012
		Higher CRP	6/103 (5.8)		

Biomarker	Visit	Baseline factor	n/m (%)	OR (95% CI)	*P* value
	Month 4	Lower CRP	34/116 (29.3)	3.86 (1.79–8.29)	0.0005
		Higher CRP	10/103 (9.7)		
	Month 16	Lower CRP	56/116 (48.3)	2.17 (1.24–3.78)	0.0065
		Higher CRP	31/103 (30.1)		
	Month 21	Lower CRP	61/116 (52.6)	2.46 (1.41–4.28)	0.0014
		Higher CRP	32/103 (31.1)		

S100A8/9 levels: lower, ≤ 3295.34 ng/mL; higher, > 3295.34 ng/mL. S100A12 levels: lower, ≤ 176.47 ng/mL; higher, > 176.47 ng/mL. CRP levels: lower, ≤ 0.2 mg/dL; higher, > 0.2 mg/dL.

Baseline is day 1 of the study. N is the number of responders in the subgroup (below or above median) at the corresponding time point; m is the number of patients in the subgroup at baseline. ORs and 95% CIs for differences are based on logistic regression model. Full model factors: baseline CRP. Missing values of efficacy response are imputed as non-responders.

CI = confidence interval; CRP = C-reactive protein; JIA-ACR100 = 100% improvement in juvenile idiopathic arthritis-American College of Rheumatology criteria; OR = odds ratio.

S100 Proteins as Potential Predictive Biomarkers of Abatacept Response in Polyarticular Juvenile Idiopathic Arthritis. Brunner HI, et al.

Supplementary Table 6. Sensitivity, specificity, and odds ratios (95% CI) of baseline levels of biomarkers (relative to median) in predicting JIA-ACR ID response.

	Abatacept alone (n = 34)					Abatacept with MTX (n = 124)				
	**OR (95% CI)**	**n**	**Sensitivity**	**Specificity**	***P* value***	**OR (95% CI)**	**n**	**Sensitivity**	**Specificity**	***P* value***
Month 3										
**Lower S100A8/9**	5.00 (0.85–29.57)	34	0.83	0.59	0.0759	2.58 (0.90–7.46)	124	0.71	0.57	0.0794
**Lower S100A8/9 + lower CRP**	5.00 (0.86–29.57)	34	0.83	0.59	0.0759	2.92 (0.97–8.85)	124	0.58	0.70	0.0579
**Lower S100A12**	2.40 (0.48–11.93)	34	0.75	0.55	0.2846	1.54 (0.57–4.19)	121	0.63	0.55	0.3998
**Lower S100A12 + lower CRP**	2.67 (0.53–13.43)	34	0.75	0.59	0.2345	1.89 (0.66–5.40)	121	0.54	0.69	0.2337
Month 4										
**Lower S100A8/9**	1.56 (0.31–7.87)	34	0.70	0.50	0.5934	6.96 (2.32–20.88)	124	0.74	0.64	0.0005
**Lower S100A8/9 + lower CRP**	1.56 (0.31–7.87)	34	0.70	0.50	0.5934	7.15 (2.27–22.53)	124	0.59	0.75	0.0008
**Lower S100A12**	3.27 (0.55–19.45)	34	0.80	0.54	0.1923	5.39 (1.90–15.32)	121	0.69	0.61	0.0016
**Lower S100A12 + lower CRP**	2.86 (0.47–17.35)	34	0.70	0.54	0.2524	5.47 (1.83–16.39)	121	0.54	0.73	0.0024
Month 16										
**Lower S100A8/9**	0.64 (0.14–2.91)	34	0.44	0.56	0.5589	4.31 (1.72–10.82)	124	0.68	0.66	0.0019
**Lower S100A8/9 + lower CRP**	0.64	34	0.44	0.56	0.5589	4.73	124	0.55	0.79	0.0021

	(0.14–2.91)					(1.76–12.70)				
**Lower S100A12**	2.06 (0.46–9.30)	34	0.67	0.56	0.3489	5.76 (2.23–14.89)	121	0.70	0.68	0.0003
**Lower S100A12 + lower CRP**	1.89 (0.41–8.61)	34	0.61	0.56	0.4130	6.75 (2.41–18.94)	121	0.57	0.81	0.0003

Lower S100A8/9: ≤ 3295 ng/mL; lower S100A12: ≤ 176 ng/mL; lower CRP: ≤ 0.2 mg/dL.

Missing values were imputed as non-responders of JIA-ACR ID.

*OR, 95% CI, and *P* values are based on univariate logistic regression model with combination of baseline S100A8/9, S100A12, and/or CRP categories as covariates. The above median category is the reference group.

CI = confidence interval; CRP = C-reactive protein; JIA-ACR ID = juvenile idiopathic arthritis-American College of Rheumatology criteria for inactive disease; MTX = methotrexate; OR = odds ratio.
